# Supplementary material for: Houttuynia cordata Thunb. Extracts Alleviate Atherosclerosis and Modulate Gut Microbiota in Male Hypercholesterolemic Hamsters
Source: Nutrients. 2024 Sep 28;16(19):3290. doi: 10.3390/nu16193290 (PMC11478543; doi:10.3390/nu16193290)
Supplement: Supplementary file 1 [file nutrients-16-03290-s001.zip › Supplemental figure legend revised.pdf]

Supplemental Figure S1. The *Houttuynia Cordat*a Thunb. extracts alter the gut microbiota structure of hamsters fed with high-cholesterol diet. (**A**) α-diversity based on Shannon index; (**B**) α-diversity based on Simpson index. (**C**) Venn diagram showing the *Houttuynia Cordata* Thunb. extracts alter the gut microbiota structure of HCD-fed hamsters. (**D**) PCoA based on Bray-Curtis method for β-Diversity showing the *Houttuynia Cordata* Thunb. extracts alter the gut microbiota structure of HCD-fed hamsters. Data represent mean ± SEM of 7-9 hamsters. Asterisks "*", "**", and "***" indicate the different levels of associations significant at $p < 0.05$, $p < 0.01$, and $p < 0.001$, respectively, by one-way ANOVA with *post hoc* Tukey's analysis. NCD, normal cholesterol diet; HCD, high cholesterol diet; HCAE, *Houttuynia Cordat*a aqueous extract; HCEE, *Houttuynia Cordata* ethanolic extract**;** L-HCAE, high cholesterol diet containing 1% *Houttuynia Cordat*a aqueous extract; H-HCAE, high cholesterol diet containing 5% *Houttuynia Cordat*a aqueous extract; L-HCEE, high cholesterol diet containing 1% *Houttuynia Cordat*a ethanolic extract; H-HCEE, high cholesterol diet containing 5% *Houttuynia Cordat*a ethanolic extract.
