# Supplementary figures and images for: Houttuynia cordata Thunb. Extracts Alleviate Atherosclerosis and Modulate Gut Microbiota in Male Hypercholesterolemic Hamsters
Source: Nutrients. 2024 Sep 28;16(19):3290. doi: 10.3390/nu16193290 (PMC11478543; doi:10.3390/nu16193290)

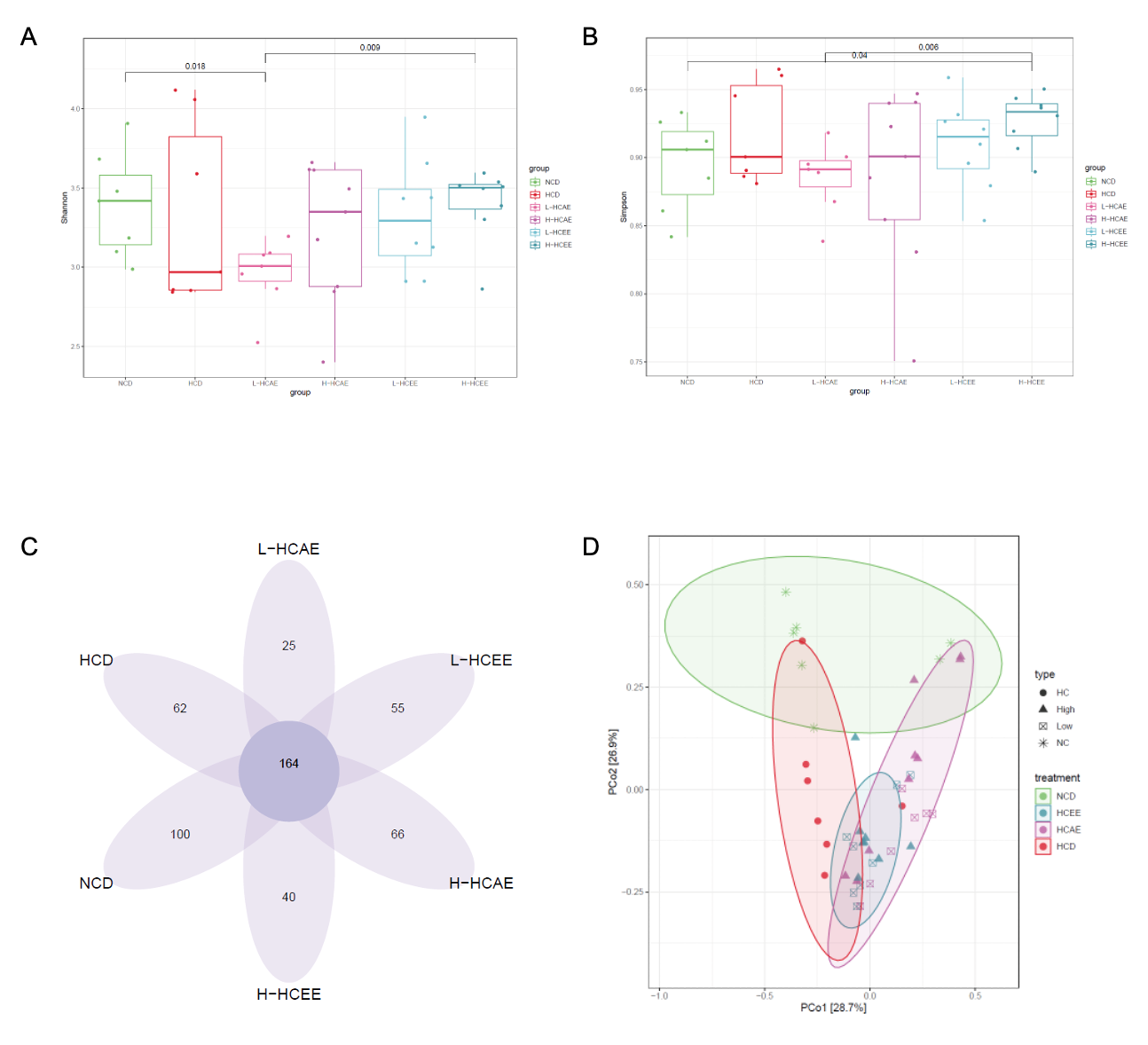

Supplement: Supplementary file 1 [file nutrients-16-03290-s001.zip › Supplemental Figure S1.png]
